# Ketone bodies for hemodynamic support in acute pulmonary embolism: a randomized, blinded, controlled animal study

**DOI:** 10.1186/s40635-025-00844-7

**Published:** 2025-12-20

**Authors:** Mads Dam Lyhne, Nigopan Gopalasingam, Kristoffer Berg-Hansen, Simone Juel Dragsbaek, Casper Homilius, Jacob Seefeldt, Jacob Valentin Hansen, Anders Dahl Kramer, Lasse Juul Christensen, Mark Stoltenberg Ellegaard, Oskar Kjærgaard Hørsdal, Andreas Overgaard, Alexander Møller Larsen, Ebbe Boedtkjer, Asger Andersen, Roni Nielsen

**Affiliations:** 1 Department of Clinical Medicine, Aarhus University, Palle Juul Jensens Boulevard 82, 8200 Aarhus N, Denmark; 2 Department of Anaesthesiology and Intensive Care, Aarhus University Hospital, Aarhus, Denmark; 3 Department of Cardiology, Aarhus University Hospital, Aarhus, Denmark; 4 Department of Biomedicine, Aarhus University, Aarhus, Denmark

**Keywords:** Right ventricular function, Pulmonary circulation, Pulmonary vascular resistance, Myography, Vasorelaxation, 3-Hydroxybutyrate

## Abstract

**Background:**

Acute pulmonary embolism (PE) is a leading cause of cardiovascular death, primarily due to abrupt increased pulmonary vascular resistance (PVR) leading to acute right ventricular (RV) failure. Ketone bodies, especially 3-hydroxybutyrate (3-OHB), have shown potential to increase cardiac output (CO) and reduce PVR in pulmonary hypertension, suggesting possible benefits in PE. We hypothesized that 3-OHB would induce pulmonary vasorelaxation and increase CO in a porcine model of acute PE.

**Methods:**

We conducted a randomized, controlled, assessor-blinded study in a porcine model of acute PE. Acute PE was induced, followed by a 3-h infusion of 3-OHB (0.22 g/kg/h, *n* = 8) or control (isovolumic saline of equimolar tonicity) (*n* = 8). Hemodynamic parameters were monitored hourly including right heart catheterization and RV pressure–volume loop acquisition. Primary outcome was the difference in CO during 3 h. Ex vivo effects on isolated pulmonary arteries were tested using wire myography.

**Results:**

Compared with control infusion, 3-OHB did not increase CO significantly (between-group difference: 0.7 [−0.2 to 1.6] L/min, *p* = 0.131). However, 3-OHB treatment lowered the PVR/systemic vascular resistance (SVR) ratio (−0.05 [−0.09; −0.01], *p* = 0.046) and increased pulmonary artery pulsatility index (5 [2–8], *p* = 0.006). Ex vivo, 3-OHB caused vasorelaxation in pre-contracted pulmonary arteries (*p* < 0.0001).

**Conclusions:**

3-OHB reduced PVR/SVR ratio, while CO was not significantly increased in a porcine model of acute PE. The present findings demonstrated potential hemodynamic effects in PE. Further studies are needed to explore the translational potential of ketone body therapy in humans with PE.

**Supplementary Information:**

The online version contains supplementary material available at 10.1186/s40635-025-00844-7.

## Background

Acute pulmonary embolism (PE) is the third most common cause of cardiovascular death due to subsequent acute right ventricular (RV) failure [1, 2]. In PE, both mechanical obstruction from the clot and secondary pulmonary vasoconstriction increase pulmonary vascular resistance (PVR) and RV afterload [3]. The healthy RV is highly sensitive to sudden increases in afterload, leading to reduced contractility and cardiac output (CO) [3–5].

Present treatments for acute PE focus on removal of the embolus, anticoagulation, or fibrinolysis to target vascular obstruction, but mortality and morbidity remain high despite optimal treatment [1, 6, 7]. Supporting the failing RV by either enhancing RV output or reducing PVR through pulmonary vasodilation could be a promising treatment approach [2]. Although a few clinical studies have explored these strategies, their effects have been limited [8–10]. This highlights the need for a novel and effective pharmacological alternative.

Ketone bodies, which are naturally produced in the liver, serve as an alternative energy source for, e.g., the heart and brain under conditions of fasting or severe illness [11, 12]. Elevated plasma levels of ketone bodies can be safely achieved through exogenous administration of 3-hydroxybutyrate (3-OHB) via intravenous or oral supplementation [13, 14]. Recent studies show that exogenous ketone body supplementation increases CO in patients with pulmonary hypertension, congestive heart failure, and acute cardiogenic shock while also reducing PVR [15–17]. Based on the hemodynamic profile of ketone bodies, exogenous ketone body supplementation may offer hemodynamic benefits in acute PE, though this has not yet been investigated.

We hypothesized that ketone body treatment would promote pulmonary vasodilation, increase CO, and decrease PVR compared to control. Thus, this study aimed to assess the hemodynamic effects of intravenous administration of the ketone body 3-hydroxybutyrate (3-OHB) in a porcine model of autologous, acute PE, and to examine its effects on isolated porcine pulmonary arteries.

## Methods

### Animals and ethics

The study was reported in accordance with the ARRIVE guidelines [18] (checklist in Supplementary) and followed Danish and international legislations and ethical standards. All investigators involved in the experiments were accredited to handle research animals. The study was approved by the Danish Animal Experiments Inspectorate (license no. 2021-15-0201-00944). 

Experimental units were female Danish slaughter pigs (crossbred of Duroc, Landrace, and Yorkshire) of approximately 60 kg. No genetic modifications were used, but animals followed the Specific Pathogen Free program. Animals arrived at the research farm 5–10 days prior to experiments to acclimatize. They were handled and observed by trained veterinary staff. Animals were housed two-by-two in pens with free access to water and were fed thrice daily.

### Study design and outcomes

**Fig. 1:**
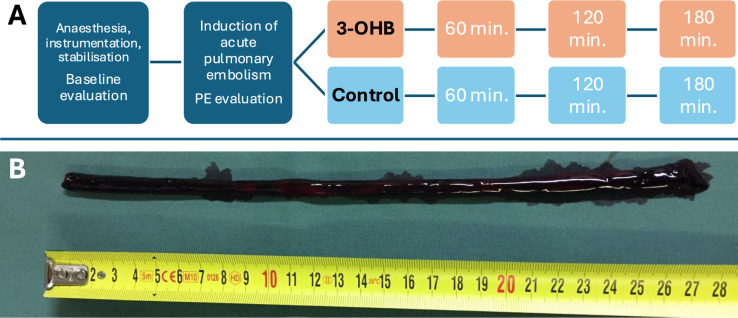
Study design and model. Legend: **a** study design. Animals were anesthetized and baseline evaluation was performed. Acute pulmonary embolism (PE) was induced until mean pulmonary arterial pressure was doubled from baseline and PE evaluation was performed. Animals were then randomized to 3 h of either 3-hydroxybutyrate (3-OHB) or control (isovolumic saline of equimolar tonicity) and evaluations were conducted hourly. **b** Example of an autologous blood clot used for the induction of central, acute PE

This study was designed as a randomized, controlled, assessor-blinded, experimental study. After induction of anaesthesia, instrumentation and baseline evaluation, each study animal was injected consecutively with autologous blood clots into the venous circulation to create a central pulmonary embolus. All animals were randomized to either receive a 3-h infusion with Na–3-OHB (0.22 g/kg/h, Gold Biotechnology Inc., St. Louis, MO, USA) or control (isovolumic NaCl of equimolar tonicity), see Fig. 1A, through a peripheral venous catheter in a superficial ear vein. 3-OHB was chosen for being readily available and feasible for both experimental and clinical use. The dose and administration form was chosen based on previously shown hemodynamic effects [15, 16] ensuring sufficient bioavailability and safe and feasible ketosis. The ketone and control solutions were prepared by an external assistant not participating in the experiments to ensure assessor-blinding. Thorough hemodynamic evaluation was performed hourly. Post-protocol, animals were euthanized by a lethal dose of pentobarbital while in deep anaesthesia.

Inclusion criteria were defined a priori and included induction of PE until doubling of baseline mean pulmonary arterial pressure (mPAP) or hemodynamic instability. Exclusion criteria included increased mPAP at baseline (>25 mmHg), signs of infection, and refractory non-sinus rhythm post-instrumentation.

Our primary outcome was the difference in CO between groups during the 3-h infusion period. Secondary endpoints included differences in PVR, mPAP, pulmonary artery pulsatility index (PAPi), RV arterial elastance (Ea), RV end-systolic elastance (Ees), mean arterial pressure (MAP), PVR/systemic vascular resistance (SVR) ratio, and lactate concentration.

### Anaesthesia, instrumentation and measurements

Animals were anaesthetized with intravenous infusion of propofol and fentanyl as previously described [19]. They were intubated and mechanically ventilated with a fraction of inspired oxygen of 30–35% to target a baseline PaO_2_ of 17–22 kPa (127–165 mmHg), PEEP of 5 cmH_2_O, tidal volume of 8 mL/kg, and a respiratory frequency adjusted before baseline to target end-tidal CO_2_ (EtCO_2_) of 5–5.5 kPa (37–41 mmHg). Afterwards, ventilator settings were unaltered. A bladder catheter was inserted, and urine output was measured at each timepoint. Animals were monitored with continuous three-point ECG.

Animals were instrumented as described in detail elsewhere [19]. In short, invasive access to the femoral artery was obtained for MAP measurements and to obtain arterial blood gases. Arterial blood samples were used for measuring circulating 3-OHB levels (FreeStyle Optium Neo, Abott, USA).

Through a large 26F sheath (Gore Dryseal) in the jugular vein, a pulmonary artery catheter (7.5F; CCOmbo, Edwards Lifescience, USA) was inserted to measure mPAP, right atrial pressure (RAP), and CO, and for mixed venous blood gasses including saturation (SvO_2_). CO was measured by the thermodilution technique as an average of three injections (10 mL isotonic glucose solution of 5 °C) with less than 10% variation. It is not possible to obtain reliable wedge pressures in this model with large, centrally located PE; therefore, another pressure catheter was advanced retrograde from the carotid artery to the left ventricle (LV) for end-diastolic pressure (EDP) measurements. PVR was calculated as (mPAP − LV EDP)/CO. SVR was calculated by the standard formula, and we calculated the PVR/SVR ratio to omit CO. PAPi was calculated as (systolic PAP − diastolic PAP)/RAP [20].

An admittance-based pressure–volume (PV) catheter (Transonic Scisense, London, Canada) was advanced through the left external jugular vein under fluoroscopic guidance to the RV, and proper positioning was controlled by fluoroscopy and optimal pressure, phase, and magnitude tracings. PV loops were recorded in LabChart 8 pro through an ADV500 control unit (Transonic Scisense) and PowerLab 8/35 (ADInstruments, Oxford, UK). PV volume calibration was performed from the thermodilution CO measurement. PV loops were recorded for 2 min at each timepoint for steady state conditions and in transient apnea (<10 s). The latter was used for post-hoc PV loop analysis with the observer blinded to treatment. A balloon was placed in the inferior vena cava to reduce preload for load-independent measurements. The transient (<10 s) preload reduction was performed thrice at each timepoint, and an average was used for analysis. PV loop analysis provided information on Ees (linear calculation), Ea, ejection fraction (EF), minimal and maximal derivative of pressure (dP/dt_min_ and dP/dt_max_, respectively), and end-systolic and end-diastolic pressures and volumes (ESP, ESV, EDP, and EDV, respectively). The right ventriculo-arterial coupling was calculated as Ees/Ea [21].

### Pulmonary embolism model

The model is based on autologous blood clots coagulating for 3 h in non-heparin-coated tubes. Each PE measured approximately 1 × 25 cm (Fig. 1B). Consecutive clots were injected through the 26F sheath to the venous circulation to cause central PE as previously described [22]. The model mimics human intermediate–high-risk PE with RV dysfunction but stable MAP [1, 19]. In accordance with the 3R framework on animal research, we did not aim for a hemodynamically unstable model with expected high mortality.

### Ex vivo* isolated pulmonary artery myography*

Lungs from eight other healthy pigs were collected from a local abattoir. Lower right lobe lung samples were submerged in ice-cold physiological saline solution (PSS) and transported to the laboratory. The PSS consisted of (in mM): 119 NaCl, 22 NaHCO_3_, 10 HEPES, 1.2 MgSO_4_, 2.82 KCl, 5.5 glucose, 1.18 KH_2_PO4, 0.03 EDTA, and 1.6 CaCl_2_ [23]. Small segments of pulmonary arteries were isolated by dissection under a stereomicroscope and mounted in isometric wire myographs (DMT 610 M, Denmark) using 40 µm thick stainless-steel wires. The PSS-filled myograph chambers were heated to 37 °C and continuously bubbled with 5% CO_2_/balance air. Following 30 min of stabilization, vessels were normalized to an internal diameter corresponding to a transmural pressure of 29.3 mmHg as previously described [16]. The normalized internal diameter was on average (±SD) 295 ± 127 µm. All arteries were initially activated by increasing the extracellular K^+^-concentration to 60 mM for two 2-min-long contractions and then by the addition of 3 µM of the thromboxane analogue U46619 for a 5-min long “initial maximal contraction”. The solution with elevated [K^+^] was produced by equimolar substitution of KCl for NaCl. Vascular responses to racemic Na–3-OHB were tested in arteries pre-contracted with U46619 to ~60% of the initial maximal contraction. Arteries were exposed to 3 and 10 mM Na–3-OHB or equimolar extra NaCl in alternating order. We report relative changes from the pre-contraction level. Solutions containing NaCl or Na–3-OHB at equimolar tonicity were prepared, heated, and adjusted to pH 7.4 as previously described [24].

### Statistics

For a priori sample size calculation, we decided that a 0.7 L/min difference in CO would be a relevant effect size in a translational study. Applying a non-paired study design, with a known standard deviation of differences of 0.44 L/min [15], a significance level of 5%, and power of 80%, we calculated a necessary sample size of *n* = 8 in each group (3-OHB vs. control) randomized 1:1. We predefined our endpoint measurements as changes from the introduction of PE to the end of study (i.e., at 3 h).

Normal distribution was evaluated by QQ plots, and normally distributed variables are presented as mean ± SD or mean ± SEM; otherwise, median [IQR]. Right-skewed non-normally distributed data were log-transformed if appropriate. The primary analysis compared the change in CO during 3-h ketone infusion compared with a 3-h control infusion. A linear mixed model was employed. Treatment and treatment-by-time interaction were defined as fixed effects, while each animal was treated as random effects. Residuals were assessed for normality and homoscedasticity, with log transformations applied if necessary. The effect size of ketone infusion compared to control is reported as the pairwise mean difference with a 95% confidence interval (CI) using Satterthwaite’s method and estimated marginal of means. Ex vivo isolated pulmonary artery experiments were compared by two-way ANOVA analysis testing for interaction. Statistical significance was defined as *p* < 0.05. All data analyses were performed using R (Version 2022.12.0, RStudio, Posit, USA) or GraphPad Prism 10.

## Results

A total of 18 animals were enrolled. Two animals died from obstructive shock following PE induction before randomisation. Accordingly, 16 animals were included in the analyses with 8 animals in each group. Induction of consecutive PE resulted in an increase in mPAP, PVR, and PVR/SVR ratio, and hypoxemia (Table 1). CO, SvO_2_, and MAP remained unaltered. Animals randomized to the control arm had higher HR and lower PaO_2_ (Table 1 and Supplementary Table 1).

**Table 1:** Baseline and after pulmonary embolism induction

	3-OHB (*n* = 8)			Control (*n* = 8)			3-OHB vs. control at PE
	Baseline	PE	*p* value	Baseline	PE	*p* value	*p* value
CO, L/min	4.0 ± 0.70	4.4 ± 0.9	0.3	4.1 ± 0.8	4.8 ± 1.3	0.3	0.8
MAP, mmHg	85 ± 11	77 ± 12	0.10	81 ± 10	85 ± 11	0.6	0.2
HR, bpm	59 ± 12	60 ± 8	0.6	59 ± 9	82 ± 18	0.010	0.024
SV, mL	70 ± 9	75 ± 19	0.3	70 ± 13	60 ± 14	0.13	0.2
RAP, mmHg	3 ± 1	5 ± 2	0.022	4 ± 2	5 ± 2	0.5	>0.9
mPAP, mmHg	14 ± 3	26 ± 6	0.002	16 ± 3	30 ± 7	0.003	0.3
PVR, WU	1.4 ± 0.8	4.0 ± 2.3	0.015	1.9 ± 1.2	5.4 ± 2.5	0.026	0.5
PVR/SVR	0.06 ± 0.03	0.23 ± 0.06	0.002	0.10 ± 0.05	0.28 ± 0.08	0.004	0.2
SvO_2_, %	59 ± 7	54 ± 8	0.10	52 ± 7	50 ± 6	>0.9	0.5
RV EF, %	63 ± 11	54 ± 13	0.2	60 ± 15	50 ± 12	0.10	0.6
Arterial PaO_2_, kPa	19 ± 2.1	15 ± 2.3	0.010	18 ± 1.9	12 ± 2.4	0.001	0.028

Hemodynamic variables at baseline and after induction of acute pulmonary embolism (PE) in animals stratified by the following treatment of either 3-hydroxybutyrate (3-OHB) or control

*CO* cardiac output, *MAP* mean arterial pressure, *HR* heart rate, *SV* stroke volume, *RAP* right atrial pressure, *mPAP* mean pulmonary arterial pressure, *PVR* pulmonary vascular resistance, *WU* Wood unit, *SVR* systemic vascular resistance, *SvO*_*2*_ mixed venous oxygen saturation, *EF* ejection fraction, *PaO*_*2*_ arterial partial pressure of oxygen

### Effects of ketone bodies

Infusion of 3-OHB increased 3-OHB plasma concentration by 1.0 ± 0.3 mmol/L, whereas control infusion changed 3-OHB concentration by 0.1 ± 0.3 mmol/L (effect size 0.8 [0.6, 1.1] mmol/L, *p* < 0.001, *n* = 8).

**Fig. 2:**
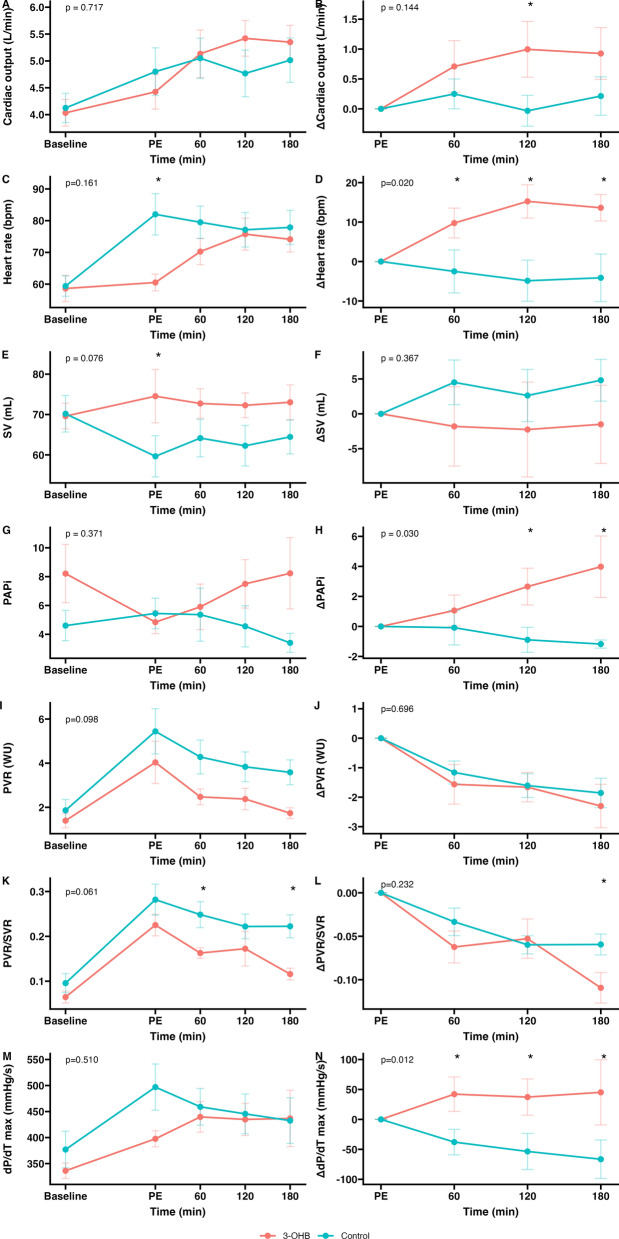
Effects of 3-OHB in acute pulmonary embolism. Effects of treatment with 3-OHB or control on key cardiopulmonary, hemodynamic variables. Cardiac output (**a**, **b**) was the primary outcome. All data are shown in absolute values from baseline through PE induction and for 3 h treatment (*left column*) and the relative changes after PE for each of the two interventions (*right column*). *p* values represent post-hoc analysis of estimated marginal means of mixed model analysis with repeated measures. Data are presented as mean ± SD. *3-OHB* 3-hydroxybutyrate, *PE* pulmonary embolism, *SV* stroke volume, *PAPi* pulmonary artery pulsatility index, *MAP* mean arterial pressure, *PVR* pulmonary vascular resistance, *SVR* systemic vascular resistance, *dP/dt max* maximal derivative of pressure

Compared with control, infusion of 3-OHB did not statistically increase the primary outcome CO (0.7 L/min [95% CI −0.2, 1.6], *p* = 0.131), while HR increased (18 [95% CI 8, 28] bpm, *p* = 0.002), and stroke volume remained stable. 3-OHB infusion increased PAPi, MAP, and RV dP/dt_max_ and decreased RAP and the PVR/SVR ratio more than control (−0.06 ± 0.03 vs. −0.11 ± 0.04, effect size −0.05 [95% CI −0.09, −0.01], *p* = 0.046, see Table 2, Fig. 2). Individual data are depicted in Supplementary Fig. 1, and absolute numbers are provided in Supplementary Table 2.

**Table 2:** Effects of ketone bodies vs. control in acute pulmonary embolism

	Effect of control infusion during 180 min	Effect of 3-OHB infusion during 180 min	Effect size (95% CI) of 3-OHB infusion compared with control infusion during 180 min	*p* value
Hemodynamics				
ΔCO, L/min	0.2 ± 0.9	0.9 ± 1.2	0.7 (−0.2, 1.6)	0.131
ΔMAP, mmHg	−4 ± 11	6 ± 12	9 (2, 17)	0.019
ΔHR, bpm	−4 ± 17	14 ± 9	18 (7, 28)	0.002
ΔSV, mL	5 ± 8	−2 ± 16	−6 (−18, 5)	0.302
ΔRAP, mmHg	0 ± 1	−2 ± 1	−2 (−3, −1)	0.001
ΔmPAP, mmHg	−5 ± 3	−5 ± 3	−1 (−3, 2)	0.654
ΔPAPi	−1 ± 1	4 ± 5	5 (2, 8)	0.006
ΔPVR, WU	−1.9 ± 1.2	−2.3 ± 1.8	−0.4 (−1.6, 0.7)	0.471
ΔSVR, WU	−2.3 ± 3.4	−2.0 ± 4.4	0.3 (−2.8, 3.4)	0.857
ΔPVR/SVR	−0.06 ± 0.03	−0.11 ± 0.04	−0.05 (−0.09, −0.01)	0.046
ΔSvO_2_, %	−1.6 ± 7.9	1.6 ± 5.5	3.1 (−2.7, 9.0)	0.316
ΔPvCO_2_, kPa	−0.3 ± 0.4	−0.4 ± 0.4	−0.1 (−0.4, 0.2)	0.545
Right ventricular function				
ΔEF, %	6 ± 4	9 ± 14	1 (−7, 10)	0.748
ΔESV, mL	5 ± 11	−17 ± 26	−18 (−33, −4)	0.022
ΔEDV, mL	38 ± 37	4 ± 22	−32 (−75, 11)	0.174
ΔEDP, mmHg	−1 ± 5	1 ± 8	2 (−3, 8)	0.410
ΔESP, mmHg	−9 ± 9	−7 ± 10	2 (−5, 10)	0.610
ΔEa, mmHg/mL	−0.6 ± 3.4	−2.4 ± 5.0	−1.6 (−4.8, 1.5)	0.341
ΔdP/dt(max), mmHg/s	−66 ± 85	45 ± 144	108 (33, 184)	0.011
ΔEes, mmHg/mL	−0.13 ± 0.18	−0.14 ± 0.21	−0.01 (−0.16, 0.14)	0.879
ΔEes/Ea	0.2 ± 0.4	0.4 ± 0.8	0.2 (−0.4, 0.9)	0.496

Difference in hemodynamic and right ventricular variables after 3 h of 3-hydroxybutyrate (3-OHB) or control in acute pulmonary embolism. Δ refers to the difference between PE induction and 3 h of treatment of 3-OHB infusion or control infusion. Effect size (difference) and 95%CI from linear mixed model analysis is provided. Differences are presented as mean ± SD, *n* = 8 for all

*CO* cardiac output, *MAP* mean arterial pressure, *HR* heart rate, *SV* stroke volume, *RAP* right atrial pressure, *mPAP* mean pulmonary arterial pressure, *PVR* pulmonary vascular resistance, *WU* Wood unit, *SVR* systemic vascular resistance, *SvO*_*2*_ mixed venous oxygen saturation, *EF* ejection fraction, *ESV* end-systolic volume, *EDV* end-diastolic volume, *Ea* arterial elastance, *dP/dt(max)* maximal first derivative of pressure, *Ees* end-systolic elastance

The changes in mPAP, PVR, RV Ea, RV Ees, and ventriculo-arterial coupling or RV EF did not differ between the two groups (Table 2; Fig. 2). Compared with control, 3-OHB treatment was associated with increased lactate and pH but lower PaO_2_ (Table 3). No significant difference in diuresis was observed between 3-OHB infusion and control (95 mL [95% CI 0, 191], *p* = 0.069).

**Table 3:** Effects of ketone bodies vs. control in acute pulmonary embolism

	Effect of control infusion during 180 min	Effect of 3-OHB infusion during 180 min	Effect size (95% CI) of 3-OHB infusion compared with control infusion during 180 min	*p* value
Arterial blood gas				
ΔLactate, mmol/L	−0.2 ± 0.2	0.2 ± 0.3	0.5 (0.3, 0.6)	<0.001
ΔPaO_2_, kPa	0.4 ± 1.5	−1.8 ± 1.0	−2.2 (−3.5, −0.9)	0.002
ΔpH	0.00 ± 0.02	0.12 ± 0.07	0.11 (0.08, 0.15)	<0.001
ΔPaCO_2_, kPa	−0.3 ± 0.4	−0.4 ± 0.4	−0.1 (−0.4, 0.2	0.545
ΔBase excess	1.0 ± 1.4	8.5 ± 1.1	9.5 (7.3, 11.7)	<0.001
ΔNa, mmol/L	6 ± 4	5 ± 1	−1 (−3, 1)	0.298

Difference in biochemical variables after 3 h of 3-hydroxybutyrate (3-OHB) or control in acute pulmonary embolism. Δ refers to the difference between PE induction and 3 h of treatment of 3-OHB infusion or control infusion. Effect size (difference) and 95% CI from linear mixed model analysis is provided. Differences are presented as mean ± SD, *n* = 8 for all

*PaO*_*2*_ arterial partial pressure of oxygen, *PaCO*_*2*_ arterial partial pressure of carbon dioxide, *Na* sodium concentration

### Ex vivo* effects of ketone bodies*

**Fig. 3:**
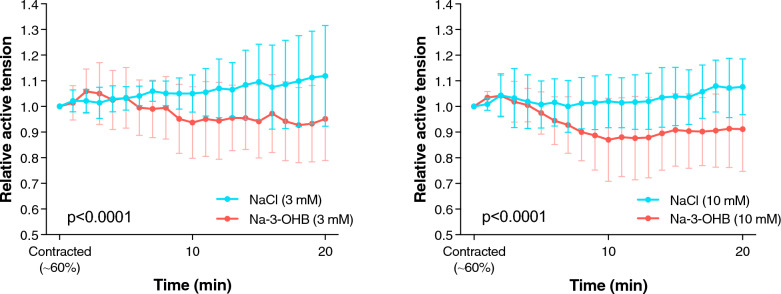
Effects of 3-OHB on pulmonary arteries. Ex vivo using isometric wire myographs, porcine pulmonary vasorelaxation was shown by 3-hydroxybutyrate (3-OHB) compared to control at both low (*left*) and higher (*right*) concentrations. Data are presented as mean ± SD

In isolated pulmonary arteries mounted for isometric tension development, 3-OHB caused pulmonary vasorelaxation at both 3 and 10 mmol/L as compared with NaCl (*p* < 0.0001 for both; Fig. 3).

## Discussion

In our experimental model mimicking intermediate-risk PE, treatment with the ketone body 3-OHB did not significantly increase CO over a 3-h treatment period compared to control. However, infusion of 3-OHB decreased RAP and PVR/SVR ratio, improved PAPi, and caused pulmonary vasorelaxation in isolated pulmonary arteries. These findings are indicative of a potential improvement in the hemodynamic state in experimental PE.

### Ketone bodies in acute pulmonary embolism

In our model of acute PE, CO did not change significantly, though the point estimate was of a clinically relevant size. It is difficult to ascribe exact causality between the individual hemodynamic parameters. One possible interpretation is HR increase due to baro-receptor reflex from systemic vasodilatation. Conversely, the observed increase in HR could also be explained by reduced intracardiac pressure with decreased chronotropic inhibition. Interpretation on chronotropic changes in the present study is further challenged by the variation between study groups, as HR increased from baseline to PE in the control group but not in the 3-OHB group, while HR increased during 3-OHB administration, but not in those who received the control treatment (Fig. 2C, D). The noted tendency of difference in CO should be interpreted in this context.

On contractility, the increase in HR potentially explains the dP/dt_max_ increase. 3-OHB treated animals exhibited reduced end-systolic volume, but, despite this, changes in RV EF did not reach statistical significance. The present findings differ from other large animal models demonstrating increased contractility of 3-OHB administration in a porcine model of cardiogenic shock [25]. Furthermore, small animal studies reported increased SV but not HR, and human studies have shown effects on both HR and SV [15, 24]. Accordingly, the effects of ketone bodies may be dependent on species, the cardiac ventricle, or the disease of investigation.

Despite being secondary endpoints, of specific interest are the improvements in PVR/SVR ratio and PAPi. The main goal during pathological conditions with elevated PVR and RV dysfunction is to reduce RV afterload without compromising the systemic circulation, which would lower MAP and RV coronary perfusion pressure [4, 5]. The present study confirmed previous findings that 3-OHB has vasodilatory properties [15, 16] which may be present in both the systemic and pulmonary vasculature. The PVR/SVR ratio decrease shows more relative pulmonary than systemic vasodilating properties of ketone bodies during a condition with PE, though the pathophysiological and auto-regulatory changes in the model may have outweighed those of 3-OHB. Second, the PAPi is an emerging index of RV dysfunction integrating forward and backward pressures from the RV, and our results show improvements above those previously reported [20, 26]. The change in PAPi was especially driven by the RAP reduction, which supports the interpretation of improved forward blood flow from the RV with reduced stagnation. Hence, 3-OHB may have beneficial hemodynamic effects during PE.

The changes in lactate levels during 3-OHB administration are well-known from our previous studies [15, 16]. The elevated lactate concentration is presumably due to metabolic energy substrate competition with resultant shuttling of glucose uptake into lactate production rather than a marker of compromised hemodynamic function. It, therefore, likely confirms 3-OHB’s uptake and metabolism.

### Pulmonary vasorelaxation by 3-OHB

The ex vivo investigation confirmed that 3-OHB has a direct vasorelaxant effect on porcine pulmonary arteries, which was translated into a reduced PVR/SVR ratio in the in vivo experiments. However, the ex vivo findings are per se not directly translational into our in-vivo studies. First, the ex vivo vessels were constricted but not prone to the complex environment of endothelial damage, hypoxia, and multifactor vasoconstriction that characterize PE-induced pulmonary vasoconstriction [3]. Second, in the applied in-vivo model of PE, we have previously shown a similar reduction in PVR over time as here presented [27]. This complicates the direct interpretation from an ex vivo to a certain in vivo phase, since 3-OHB may have been administered during a phase of concomitant auto-regulatory pulmonary vasorelaxation not occurring in an ex vivo setup with isolated pulmonary vessels. Although the present study was not designed for that purpose, we speculate that ketone body treatment has potential by being more efficient in the later phase of acute PE when auto-regulatory vasomotor responses have been exhausted.

### Clinical relevance

Our results origin from an experimental study and cannot be directly translated to humans, though we have shown that 3-OHB reduces PVR and increases CO in patients with pulmonary hypertension, including chronic thromboembolic pulmonary hypertension [16]. In clinical settings, most PE-related treatments come with a high risk of severe side effects, including haemorrhage and death from anticoagulation or fibrinolysis, as well as arrythmias and increased myocardial oxygen demand from inotropes and vasopressors. There is a clinical need for a safe and effective supportive treatment in acute PE. 3-OHB has shown improvements in patients with cardiogenic shock [17] emphasising that ketone bodies have clinical relevance in shock of different aetiologies, and the present study was conducted to extend the knowledge on the potential utility of ketone bodies. Despite our neutral primary outcome, we believe ketone bodies could be further explored in the settings of acute PE.

### Limitations

This study has some limitations to consider. First, though it is an animal study with affected clinical translation, we observed an effect size in the animals comparable to the one used in the sample size calculation from human data but with higher-than-expected variation. The model mimicked intermediate–high-risk PE with maintained MAP, though normotensive shock with increased pulmonary pressure and reduced RV function has been described in PE [28]. This may limit the translation of the present study results to patients with such PE condition. Second, despite the randomized design, it appeared that the control animals were more severely affected by acute PE than 3-OHB-treated animals, though there was no difference in CO at baseline. This leaves more therapeutic (or passive) potential for improvement, possibly limiting the difference in effect size between the two groups. Hence, we cannot exclude that the response to the infusions may have differed, which calls for caution during interpretation of any of our findings. Third, other ketone supplements could have been investigated. However, due to our previous studies and knowledge regarding the hemodynamic effects of 3-OHB, we chose to assess this ketone compound in the present study. Other evaluation methods, e.g., echocardiography, could perhaps have provided additional insight but were omitted due to the shape of the porcine thorax making it difficult to achieve reliable acoustic windows. Similarly, biomarkers or 12-point ECG were not included. Fourth, oxygen is a pulmonary vasodilator [19], and we aimed for a higher PaO_2_ target to avoid severe hypoxemia mimicking a clinical approach. We cannot exclude that the reduced vasomotor tone from supplemental oxygen may have limited the therapeutic signal from 3-OHB driving results towards the null. Fifth, ketone bodies are known to improve cardiac function and remodelling over time [11], whereas we only investigated acute effects of 3-OHB treatment. Finally, pH increased during 3-OHB infusion probably because cellular uptake and metabolism of 3-OHB are coupled with H^+^ uptake and consumption. However, the observed change in pH is not expected to cause the changes in hemodynamic measures [15, 16].

## Conclusion

In a porcine model of acute PE, administration of the ketone body 3-hydroxybutyrate did not significantly increase CO but improved PAPi. Further research is warranted to investigate if ketone bodies are effective in more stable phases of increased PVR, and if the effects are similar in patients with acute PE.

## Supplementary Information

Additional file 1.

Additional file 2.

Additional file 3.

## Data Availability

The data sets generated during and/or analysed during the current study are available from the corresponding author on reasonable request.

## Abbreviations

3-OHB: 3-Hydroxybutyrate
CO: Cardiac output
dP/dt_max_: Maximal first derivative of pressure
dP/dt_min_: Minimal first derivative of pressure
Ea: Arterial elastance
Ees: End-systolic elastance
EF: Ejection fraction
MAP: Mean arterial pressure
mPAP: Mean pulmonary arterial pressure
PaO_2_: Arterial partial pressure of oxygen
PAPi: Pulmonary artery pulsatility index
PE: Pulmonary embolism
PEEP: Positive end-expiratory pressure
PVR: Pulmonary vascular resistance
RAP: Right atrial pressure
RV: Right ventricle
SV: Stroke volume
SvO_2_: Mixed venous oxygen saturation
SVR: Systemic vascular resistance
