# Supplementary figures and images for: Ketone bodies for hemodynamic support in acute pulmonary embolism: a randomized, blinded, controlled animal study
Source: Intensive Care Med Exp. 2025 Dec 20;13:133. doi: 10.1186/s40635-025-00844-7 (PMC12717324; doi:10.1186/s40635-025-00844-7)

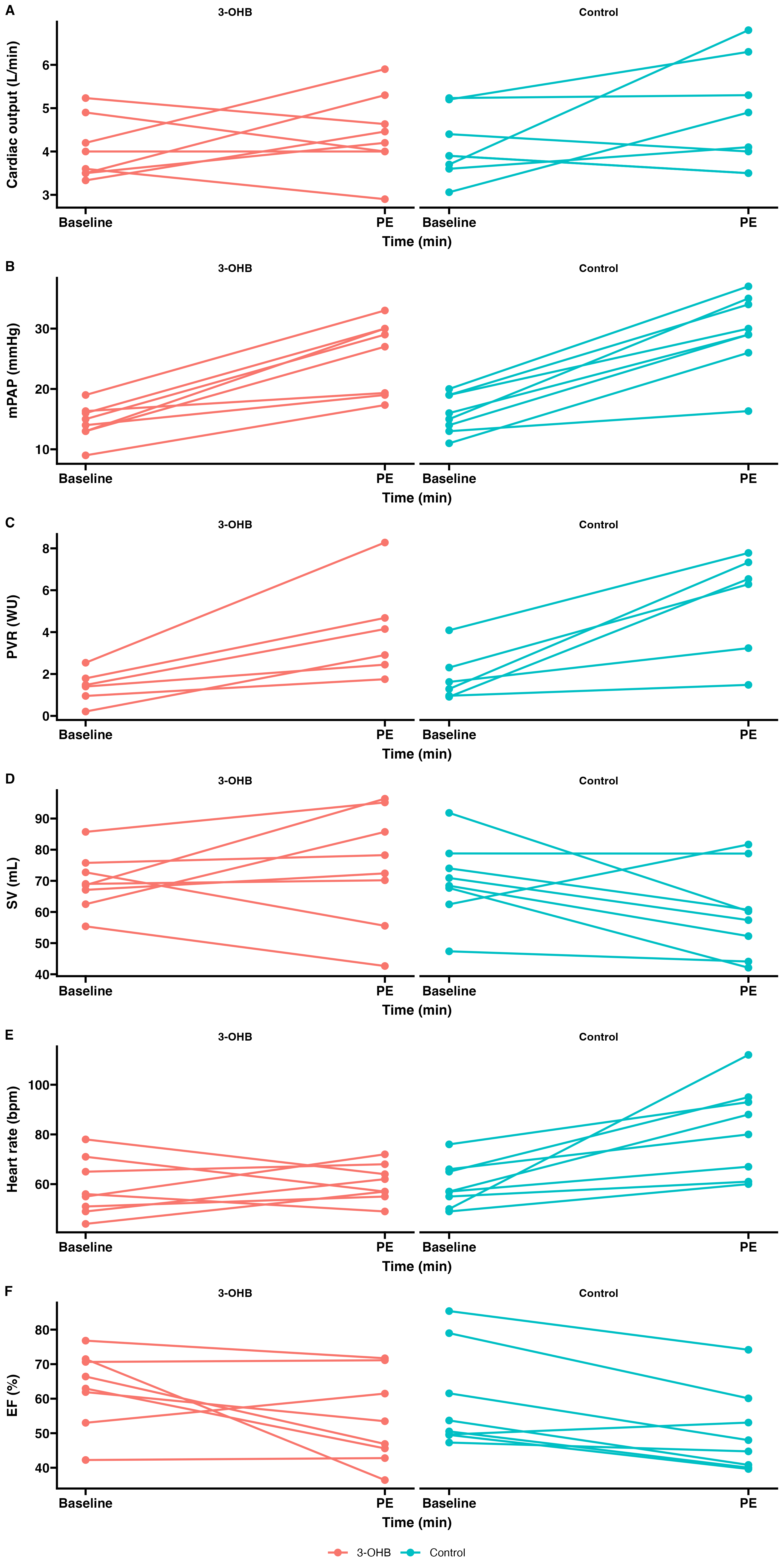

Supplement: Supplementary file 1 — Additional file 1. [file 40635_2025_844_MOESM1_ESM.png]
