# Supplementary material for: Ketone bodies for hemodynamic support in acute pulmonary embolism: a randomized, blinded, controlled animal study
Source: Intensive Care Med Exp. 2025 Dec 20;13:133. doi: 10.1186/s40635-025-00844-7 (PMC12717324; doi:10.1186/s40635-025-00844-7)
Supplement: Supplementary file 2 — Additional file 2. [file 40635_2025_844_MOESM2_ESM.docx]

**Table S1: Hemodynamic measures at baseline and following pulmonary embolism**

|  | **3-OHB (n=8)** | | | **Control (n=8)** | | |
| --- | --- | --- | --- | --- | --- | --- |
|  | Baseline | PE | p-value | Baseline | PE | p-value |
| RAP, mmHg | 2.6 ± 1.4 | 4.9 ± 1.7 | 0.022 | 4.1 ± 1.6 | 4.8 ± 2.1 | 0.5 |
| SVR, WU | 21 ± 4.9 | 17 ± 5.3 | 0.2 | 19 ± 4.3 | 18 ± 5.9 | 0.8 |
| Stroke volume, mL | 70 ± 9.0 | 75 ± 19 | 0.3 | 70 ± 13 | 60 ± 14 | 0.13 |
| PAPi | 8.2 ± 5.7 | 4.8 ± 2.3 | 0.3 | 4.6 ± 3.0 | 5.4 ± 3.0 | 0.3 |
| Volume end-systole, mL | 57 ± 30 | 73 ± 25 | 0.038 | 51 ± 21 | 58 ± 21 | 0.4 |
| Volume end-diastole, mL | 132 ± 48 | 152 ± 59 | 0.8 | 117 ± 57 | 104 ± 39 | 0.7 |
| Ea, mmHg/mL | 0.47 ± 0.48 | 2.6 ± 4.7 | 0.065 | 0.77 ± 0.50 | 2.0 ± 2.4 | 0.083 |
| dp/dT max, s | 336 ± 42 | 398 ± 44 | 0.028 | 377 ± 99 | 497 ± 125 | 0.065 |
| Ees, mmHg/mL | 0.31 ± 0.16 | 0.57 ± 0.32 | 0.083 | 0.41 ± 0.30 | 0.70 ± 0.39 | 0.3 |
| Ees/Ea | 0.90 ± 0.59 | 0.68 ± 0.64 | 0.3 | 0.60 ± 0.28 | 0.62 ± 0.66 | 0.5 |
| Lactate, mmol/L | 0.90 ± 0.29 | 0.74 ± 0.22 | 0.2 | 1.2 ± 0.42 | 0.80 ± 0.28 | 0.072 |
| pH | 7.5 ± 0.02 | 7.4 ± 0.07 | 0.002 | 7.5 ± 0.02 | 7.4 ± 0.03 | 0.003 |
|  | | | | | | |
|  | | | | | | |
