# Supplementary material for: Ketone bodies for hemodynamic support in acute pulmonary embolism: a randomized, blinded, controlled animal study
Source: Intensive Care Med Exp. 2025 Dec 20;13:133. doi: 10.1186/s40635-025-00844-7 (PMC12717324; doi:10.1186/s40635-025-00844-7)
Supplement: Supplementary file 3 — Additional file 3. [file 40635_2025_844_MOESM3_ESM.docx]

**Supplementary Table 1:**

|  | **Mean after 180 minutes of control infusion** | **Mean after 180 minutes of 3-OHB infusion** | **Effect size (95% CI) of 3-OHB infusion compared with control infusion during 180 min** | **p-value** |
| --- | --- | --- | --- | --- |
| **Hemodynamics** | | | | |
| **CO, L/min** | 5.0 ± 1.2 | 5.3 ± 0.9 | 0.7 (−0.2, 1.6) | 0.131 |
| **MAP, mmHg** | 81 ± 14 | 83 ± 9 | 9 (2, 17) | 0.019 |
| **HR, bpm** | 78 ± 15 | 74 ± 11 | 18 (7, 28) | 0.002 |
| **SV, mL** | 64 ± 12 | 73 ± 12 | −6 (−18, 5) | 0.302 |
| **RAP, mmHg** | 5 ± 2 | 3 ± 2 | −2 (−3, −1) | 0.001 |
| **mPAP, mmHg** | 25 ± 5 | 20 ± 4 | −1 (−3, 2) | 0.654 |
| **PAPi** | 3 ± 2 | 8 ± 7 | 5 (2, 8) | 0.006 |
| **PVR, WU** | 3.6 ± 1.4 | 1.7 ± 0.6 | −0.4 (−1.6, 0.7) | 0.471 |
| **SVR, WU** | 15.6 ± 3.1 | 15.2 ± 2.2 | 0.3 (−2.8, 3.4) | 0.857 |
| **PVR/SVR** | 0.22 ± 0.06 | 0.12 ± 0.03 | −0.05 (−0.09, −0.01) | 0.046 |
| **SvO₂, %** | 48.5 ± 9.2 | 55.2 ± 5.9 | 3.1 (−2.7, 9.0) | 0.316 |
| **PvCO₂, kPa** | 7.7 ± 0.5 | 7.2 ± 0.5 | −0.1 (−0.4, 0.2) | 0.545 |
| **Right ventricular function** | | | | |
| **EF, %** | 57 ± 15 | 61 ±.14 | 1 (−7, 9) | 0.828 |
| **ESV, mL** | 57 ± 19 | 57 ± 7 | −17 (−32, −2) | 0.034 |
| **EDV, mL** | 134 ± 67 | 155 ± 70 | −29 (−72, 14) | 0.222 |
| **EDP, mmHg** | 9 ± 6 | 10 ± 7 | 2 (−3, 8) | 0.410 |
| **ESP, mmHg** | 40 ± 5 | 37 ± 12 | 2 (−5, 10) | 0.610 |
| **Ea, mmHg/mL** | 1.7 ± 2.2 | 0.5 ± 0.4 | −1.7 (−4.8, 1.5) | 0.334 |
| **dP/dt(max), mmHg/s** | 433 ± 116 | 437 ± 143 | 107 (31, 182) | 0.012 |
| **Ees, mmHg/mL** | 0.57 ± 0.36 | 0.43 ± 0.21 | −0.01 (−0.16, 0.14) | 0.879 |
| **Ees/Ea** | 0.8 ± 1.0 | 1.0 ± 0.4 | 0.2 (−0.4, 0.8) | 0.549 |
| **Arterial blood gas** | | | | |
| **Lactate, mmol/L** | 0.6 ± 0.2 | 1.0 ± 0.3 | 0.4 (0.3, 0.6) | <0.001 |
| **PaO₂, kPa** | 12.9 ± 2.8 | 13.6 ± 2.0 | −2.2 (−3.5, −0.9) | 0.002 |
| **pH** | 7.40 ± 0.04 | 7.53 ± 0.03 | 0.11 (0.08, 0.15) | <0.001 |
| **PaCO₂, kPa** | 7.7 ± 0.5 | 7.2 ± 0.5 | −0.1 (−0.4, 0.2) | 0.545 |
| **St. bicarbonate** | — | — | — | — |
| **Base excess** | 5.2 ± 1.1 | 15.5 ± 1.8 | 9.5 (7.3, 11.7) | <0.001 |
| **Na, mmol/L** | 144 ± 5 | 142 ± 2 | −1 (−3, 1) | 0.298 |
